# Targeting the epidermal growth factor receptor (EGFR/ErbB) for the potential treatment of renal pathologies

**DOI:** 10.3389/fphar.2024.1394997

**Published:** 2024-08-21

**Authors:** Mohamed Tawengi, Yazan Al-Dali, Abdelaziz Tawengi, Ibrahim F. Benter, Saghir Akhtar

**Affiliations:** ^1^ College of Medicine, QU Health, Qatar University, Doha, Qatar; ^2^ Faculty of Pharmacy, Final International University, Kyrenia, Cyprus

**Keywords:** epidermal growth factor receptor, ErbB, diabetic nephropathy, hypertensive nephropathy, glomerulonephritis, chronic kidney disease, renal fibrosis, acute kidney injury (AKI)

## Abstract

Epidermal growth factor receptor (EGFR), which is referred to as ErbB1/HER1, is the prototype of the EGFR family of receptor tyrosine kinases which also comprises ErbB2 (Neu, HER2), ErbB3 (HER3), and ErbB4 (HER4). EGFR, along with other ErbBs, is expressed in the kidney tubules and is physiologically involved in nephrogenesis and tissue repair, mainly following acute kidney injury. However, its sustained activation is linked to several kidney pathologies, including diabetic nephropathy, hypertensive nephropathy, glomerulonephritis, chronic kidney disease, and renal fibrosis. This review aims to provide a summary of the recent findings regarding the consequences of EGFR activation in several key renal pathologies. We also discuss the potential interplay between EGFR and the reno-protective angiotensin-(1–7) (Ang-(1–7), a heptapeptide member of the renin-angiotensin-aldosterone system that counter-regulates the actions of angiotensin II. Ang-(1–7)-mediated inhibition of EGFR transactivation might represent a potential mechanism of action for its renoprotection. Our review suggests that there is a significant body of evidence supporting the potential inhibition of EGFR/ErbB, and/or administration of Ang-(1–7), as potential novel therapeutic strategies in the treatment of renal pathologies. Thus, EGFR inhibitors such as Gefitinib and Erlinotib that have an acceptable safety profile and have been clinically used in cancer chemotherapy since their FDA approval in the early 2000s, might be considered for repurposing in the treatment of renal pathologies.

## 1 Introduction

**FIGURE 1:**
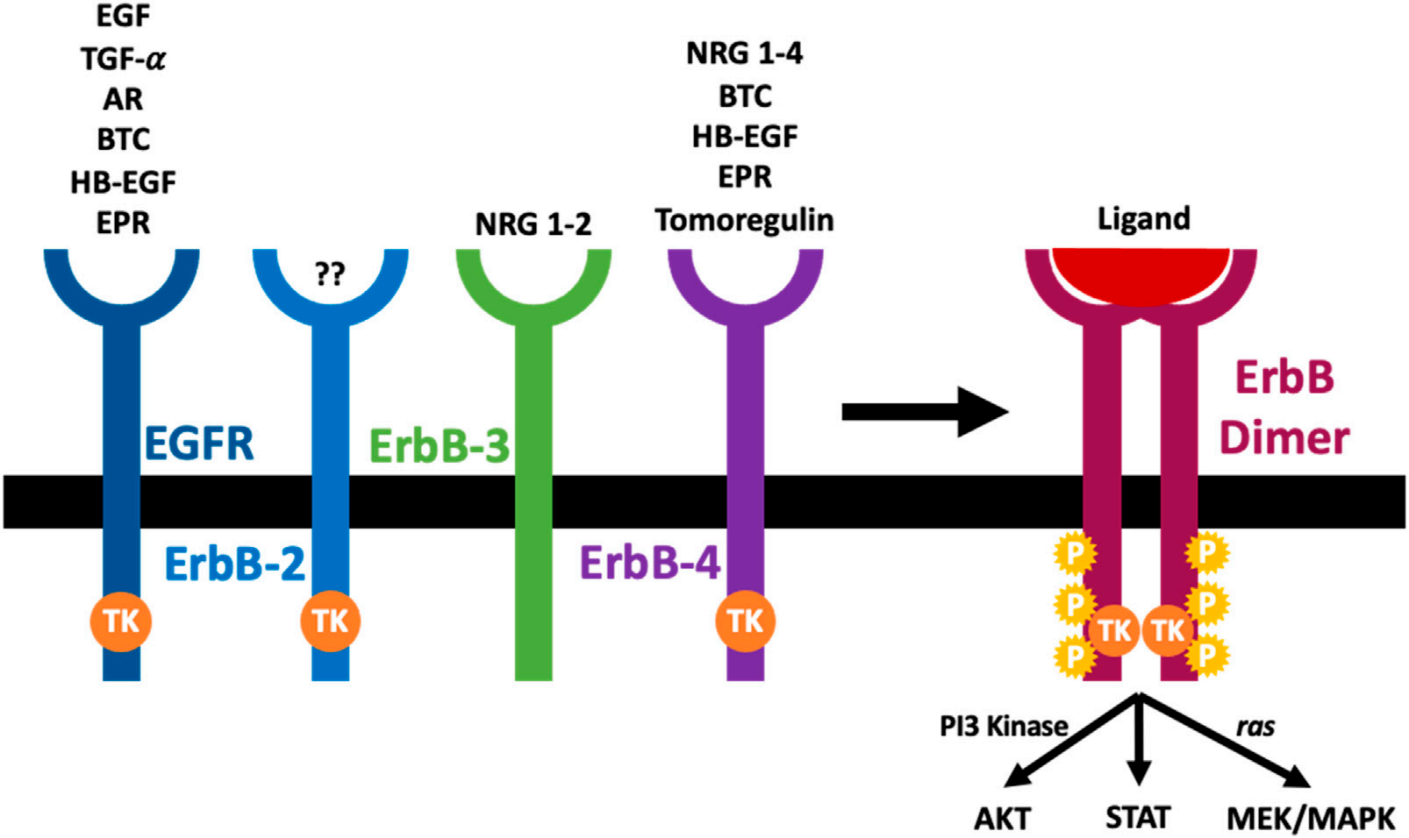
The arrangement and cognate ligands of the ErbB family of receptors. An ErbB receptor is composed of an extracellular domain that binds specific ligands, a transcellular component, and an intracellular domain with a tyrosine kinase activity (TK). The ErbB-2 is not known to have any ligand, while the ErbB-3 is known to lack kinase activity. Once a ligand binds the extracellular domain of ErbB receptors, it induces dimerization of the receptor (homodimerization or heterodimerization), tyrosine kinase activation (TK), trans-phosphorylation (P) and initiation of downstream signaling cascades (see main text for further details).

Epidermal growth factor receptor (EGFR), also called ErbB1/HER1, is the prototype of the four-membered EGFR receptor tyrosine kinases family that also comprises ErbB-2 (Neu, HER2), ErbB-3 (HER3), and ErbB-4 (HER4) (Kumagai et al., 2021; Sharifi et al., 2021; Shraim et al., 2021; Shaban et al., 2023; Popović et al., 2024) (see also Figure 1). Through their regulation by several different receptor ligands and ability to form multiple homo- and heterodimer combinations, signalling via the ErbB family members yields a diverse array of signalling outputs that regulate key cellular functions (Lemmon and Schlessinger, 2010; Wee and Wang, 2017; Shaban et al., 2023; Popović et al., 2024). All members of the EGFR family have an N-terminal extracellular ligand-binding domain, a cytoplasmic tyrosine kinase catalytic domain linked by a single transmembrane helix, and a C-terminal tail with multiple phosphorylation sites. Although they share a general architecture, the extracellular domain of each receptor is quite different, resulting in variability of ligand specificity and several biological responses (Tang et al., 2013a; Shaban et al., 2023; Popović et al., 2024). In contrast, a delicate conformation of the tyrosine kinase domain makes up the intracellular domain of the different ErbB receptors. The kinase domain of ErbB3 is an exception containing substitutions of important amino acids rendering it without kinase activity (Normanno et al., 2005).

The EGFR family can interact with various ligands (see Figure 1). Based on their ability to bind to the different ErbB receptors, the epidermal growth factor (EGF)-related growth factors are split into three different groups. The first group has EGF, transforming growth factor-α (TGF-α), and amphiregulin (AR), and they bind the EGF receptor; the second group is made up of several growth factors which can bind both EGFR and ErbB4, and they include betacellulin (BTC), heparin-binding growth factor (HB-EGF) and epiregulin (EPR). The final group comprises tomoregulin and the neuregulins (NRGs) or heregulins (HRG) which are split into two subclasses depending on their affinity to ErbB-3 and ErbB-4 (NRG-1 and NRG-2) or merely ErbB-4 (NRG-3 and NRG-4). All EGF-ligand family members are produced as membrane-tethered precursors that can be cleaved by specific metalloproteases to shed the soluble bioactive form from the cell surface (Harris et al., 2003; Rayego-Mateos et al., 2018a, 2018b; Shraim et al., 2021; Shaban et al., 2023; Popović et al., 2024). Until now, no ligand has been identified with a high affinity for ErbB-2 (Normanno et al., 2005). EGFR activation without direct ligand-stimulated interaction with the receptor ectodomain is known as transactivation (indirect activation). Several ‘transactivating” molecules exist, and they are generally classified into activators of G protein-coupled receptors (e.g., Angiotensin II (Ang II) and endothelin 1 (ET-1), cytokines (e.g., TNFA, TWEAK, and other TNF receptor family proteins), growth factors (e.g., TGFB and other proteins, including TPA and LPA), integrins, ion channels, and other physical stimuli (Rayego-Mateos et al., 2018b; Shraim et al., 2021; Shaban et al., 2023; Popović et al., 2024).

Under unstimulated conditions, EGFR is in a state of auto-inhibition and is unable to dimerize on the plasma membrane. Physiological EGFR signaling involves two structurally coupled allosteric activation events across the membrane: an extracellular ligand-dependent extracellular domain dimerization followed by an intracellular dimer-dependent tyrosine kinase activation. Ultimately, ligand binding promotes receptor dimerization, which regulates a chain of structural readjustments which get transported to the intracellular domain to allow for the formation of asymmetric dimers between two juxtaposed catalytic domains (Sigismund et al., 2018; Tsai and Nussinov, 2019). Downstream effects include the activation of several signaling pathways including the extracellular signal-regulated kinase (ERK1/2) pathway, the Janus kinase (JAK)/signal transducers and activators of transcription (STAT) pathway, and the phosphoinositide-3-kinase (PI3K)/AKT (protein kinase B) pathway. Indeed, EGFR stimulation activates a cascade of nuclear signaling that controls the function of different transcription factors including c-JUN, c-FOS, c-MYC, and NF-κB that control gene transcription. The net effects of such molecular events are associated with multiple functions including cell survival, proliferation, differentiation, and migration (Tang et al., 2013a; Rayego-Mateos et al., 2018a; 2018b; Shaban et al., 2023; Popović et al., 2024).

**FIGURE 2:**
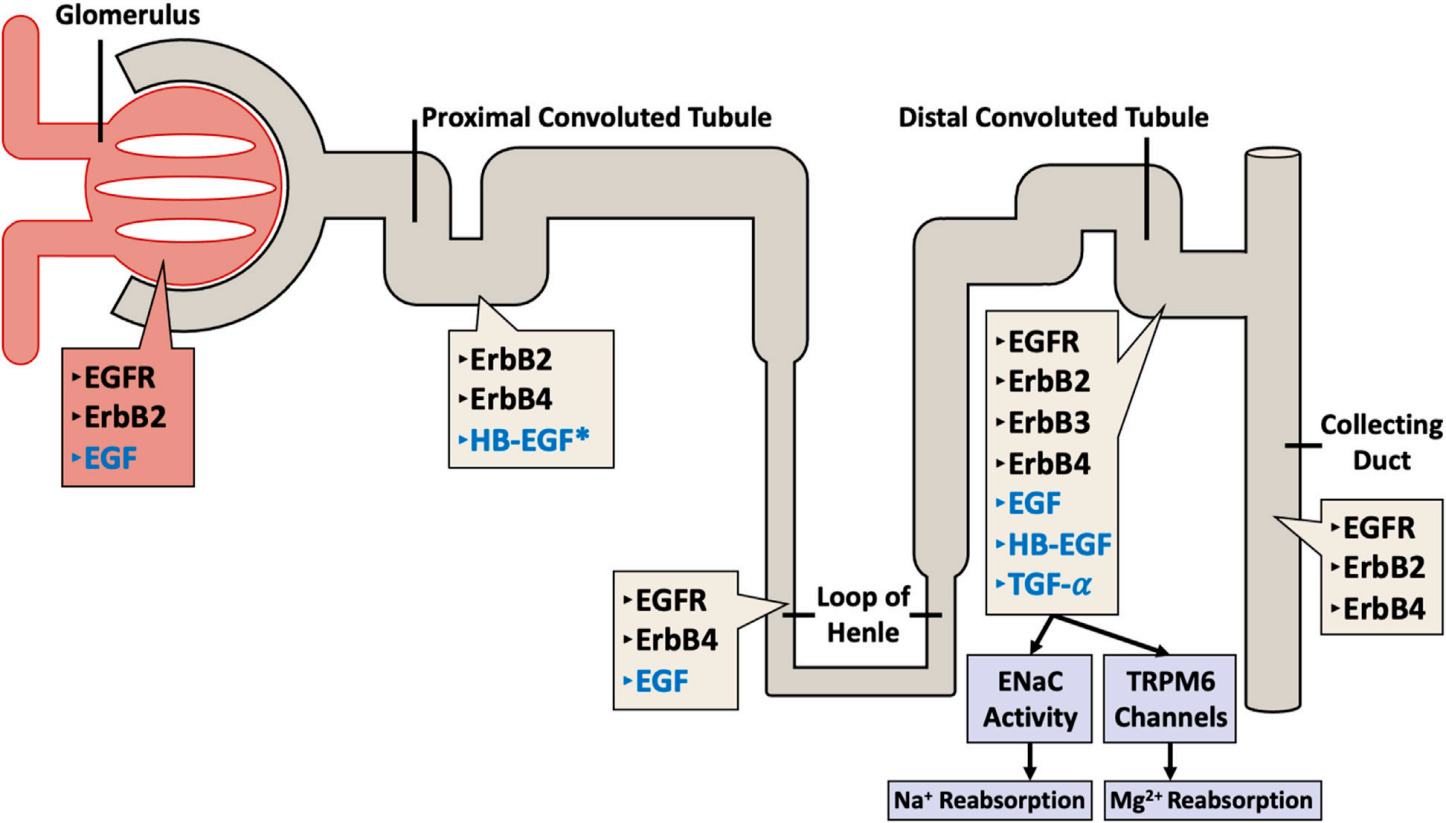
Distribution of ErbB family of receptors (in black text) with their corresponding ligands (in blue text) throughout the nephron. The different receptors and their ligands are differentially expressed along the various segments of the nephron (including the glomerulus, proximal convoluted tubule, loop of Henle, distal convoluted tubule, and the collecting duct) in a human adult kidney. Note the exception: HB-EGF expression data is from rat kidneys (asterisked).

In the kidney, all four EGFR/ErbB receptors, as well as their ligands are expressed along the length of the nephron (see also Figure 2). The receptors are mostly localized at the distal tubular cells, the connecting tubules, and the collecting ducts, and most of the ligands for these receptors are formed locally in the kidneys (Staruschenko et al., 2013) implying a possible local paracrine-autocrine function. Recent evidence suggests that kidney EGFR expression and activation after injury might also be dependent on gender (Harris, 2021).

The EGFR/ErbB pathway plays an important physiological or beneficial role in kidney development and function. Indeed, EGFR knockout mice suffer from impaired epithelial cell development in multiple organs, including the kidney (Threadgill et al., 1995; Sibilia et al., 2007). However, limited *in vivo* functional information can be obtained from knockout (KO) mice of any of the four EGFR/ErbB receptors because of the perinatal lethality of EGFR deficient mice, with embryos typically only surviving a few weeks due to, amongst others, impaired cardiovascular, skin, neuronal and gastrointestinal development (for reviews see also Sibilia et al., 2007; Sanchez-Soria and Camenisch, 2010; Shraim et al., 2021). However, knockouts of their ligands or conditional and/or site-specific (e.g., podocyte-specific) deletion of EGFR family of receptors as well as pharmacological inhibition studies have revealed that these RTKs play a detrimental role in the development and progression of different renal pathologies (Harskamp et al., 2016; Rayego-Mateos et al., 2018b; Melenhorst et al., 2008) (to be discussed further below). Thus, this potential dual (or “Jekyl and Hyde”) role of EGFR family members in the kidneys, whereby there are necessary/beneficial for development and tissue repair, but sustained overactivity is detrimental in mediating pathologies, is akin to that observed in the cardiovascular system (Akhtar and Benter, 2013; Shraim et al., 2021).

## 2 Glomerulonephritis

Glomerulonephritis (GN) is an inflammation within the glomerulus and other parts of the kidney resulting from a variety of immune-mediated disorders or infections (Chadban and Atkins, 2005; Satoskar et al., 2020; Pesce et al., 2021; Zhan et al., 2024). The incidence rate of GN worldwide is estimated to be between 0.2 and 2.5/100,000/year (McGrogan et al., 2011), with the prevalence being 306/100,000 in the US (Wetmore et al., 2016). EGFR ligands and receptor signaling have been implicated in the development of several forms of GN (see Table 1 for summary). One of these disorders in GN is the Rapid Progressive Glomerulonephritis (RPGN) which is the formation of crescent-shaped necrotizing lesions within the glomeruli with accumulation of CD4^+^ T cells and macrophages as well as proliferation of intrinsic glomerular cells, resulting in a swift decline of renal function. Increased EGFR signalling is known to promote glomerular damage and renal failure in rapidly progressive crescentic glomerulonephritis (Bollée et al., 2011). The detrimental role of EGFR in GN is supported by a study in a type 2 diabetes mouse model where EGFR inhibition with Erlotinib (a selective, clinically used EGFR inhibitor) resulted in decreased albuminuria and glomerulosclerosis, attenuated podocyte loss, and reduced renal fibrosis (Li et al., 2018).

**TABLE 1:** Summary of selected studies highlighting the role of EGFR signalling in glomerulonephritis and associated pathologies.

Pathology	Model	Methodology	Findings/Results/Conclusion relevant to EGFR	Study Reference
Glomerulonephritis	T2D Mice	EGFR Inhibitor (Erlotinib)	Less albuminuria and glomerulosclerosis in Erlotinib-treated mice	Li et al. (2018b)
Glomerulonephritis	Angiotensin II infusion Mice	Endothelial HB-EGF deletion	Less albuminuria and glomerulosclerosis in model mice	Zeng et al. (2016)
Lupus Nephritis	Fcgr2b−/− mice	TNF-α and HB-EGF/EGFR signaling blockage	TNF-α and EGFR signaling mediates Lupus Nephritis	Qing et al. (2018)
Lupus Nephritis	LN patients	Kidney miRNA pattern controlled by HER-2	Urinary HER-2 was enhanced in LN along with HER-2 overexpression	Costa-Reis et al. (2015)
Mesangial proliferative glomerulonephritis	Mesangial glomerulonephritis -induced mice	EGFR Inhibitor (Erlotinib	Pathology early events were avoided using Erlotinib	Rintala et al. (2016)
Focal Segmental Glomerulosclerosis	Cultured podocytes	Gain of mutation in TRPC6 to induce FSGS	ERK1/2 are activated	Chiluiza et al. (2013)
Glomerulonephritis	AREG −/− and myeloid cell-specific EGFR deficiency mice	Intraperitoneal injection of nephrotoxic sheep serum inducing Nephrotoxic nephritis (NTN)	AREG worsens glomerulonephritis by recruiting and activating myeloid cells	Melderis et al. (2020)

Other studies have also implicated EGFR ligands in mediating GN pathology (see also Table 1). For example, several studies have shown that HB-EGF, a ligand for EGFR, appears to be a key player in mediating pathological changes in the glomerulus resulting in glomerulonephritis (Feng et al., 2000; Tang et al., 2013a; Tang et al., 2013b; Hénique et al., 2013). Thus, HB-EGF ligand-activated EGFR signalling appears critical in mediating GN. Interestingly, a recent study showed that a subset of EGF-deficient mice developed crescentic glomerulonephritis due to upregulated HB-EGF/EGFR activation in glomeruli (Zeid et al., 2022) implying also that physiological levels of EGF, as well as HB-EGF, act as reno-protective agents against the development of glomerulonephritis.

Additionally, it has been shown that Ang II-mediated shedding of EGF-like ligands such as HB-EGF and TGF-alpha, can transactivate EGFR signalling in the kidney that leads to several renal pathologies (Lautrette et al., 2005; Chen et al., 2006; Zeng et al., 2016). For example, deletion of endothelial HB-EGF in mice reduced Ang II infusion-related renal damage by decreasing albuminuria, glomerulosclerosis, and podocyte injury, whilst maintaining the integrity of the endothelium (Zeng et al., 2016). These studies imply important crosstalk between the Renin-Angiotensin-Aldosterone system (RAAS) of which Ang II is the main component, and EGFR signalling (see also discussion on EGFR/ErbB and Ang-(1–7) crosstalk below in Section 9).

To further study the role of EGFR ligands in glomerular pathology, a recent study investigated the role of amphiregulin (AREG), an EGF-like ligand that can possess both pro and anti-inflammatory properties (Melderis et al., 2020). In a mouse model of glomerulonephritis, it was found that upregulation of AREG plays an important proinflammatory role, by enhancing myeloid cell responses and directing the macrophages toward a pro-inflammatory M1 phenotype whereas AREG knockout mice displayed considerable improvement in disease. Importantly, in the myeloid cells, the selective deletion of EGFR signaling was enough to be protective against nephritis (Melderis et al., 2020) (See also Table 1). These authors also found a strong upregulation of renal AREG expression in human crescentic glomerulonephritis (Melderis et al., 2020) and thereby suggesting that AREG/EGFR axis might represent a new therapeutic target for patients with acute glomerulonephritis.

Another disorder that could lead to progressive renal dysfunction is Lupus Nephritis (LN). A recent study suggested that permanent kidney injury occurs due to iRhom2/ADAM17-dependent TNF-α and EGFR signaling through activation of the inactive rhomboid two (iRhom2), the regulator of disintegrin and metalloprotease 17 (ADAM17) (Qing et al., 2018). In addition, urinary HER-2/ErbB2, that is involved in mesangial cell proliferation, was increased in LN and correlated with disease activity (Costa-Reis et al., 2015). Thus, a potentially novel strategy to reduce cell proliferation and damage in LN could be via blockade of both EGFR and ErbB2 signalling.

Amongst the spectrum of glomerular diseases, mesangial proliferative glomerulonephritis (MPGN) is another disorder that could result in end-stage renal disease. Treatment of mice bearing Thy1.1-antibody-induced MPGN with Erlotinib, a selective EGFR inhibitor, significantly attenuated the glomerular inflammatory response, mesangial cell proliferation, matrix accumulation and further, preserved renal function (Rintala et al., 2016). These results suggested that EGFR inhibition could effectively prevent the initial or early-phase pathological events involved in the development of MPGN and thus, may represent a potential therapeutic option in halting the progression of GN. Supportive of the role of EGFR signaling in glomerulonephritis is the finding that extracellular signal-regulated kinases 1/2 (ERK1/2), which are downstream signalling effectors for EGFR, are also activated in focal segmental glomerulosclerosis that was induced by a gain of mutation in the transient receptor potential C6 (TRPC6) (Chiluiza et al., 2013). Thus, EGFR activation and subsequent signalling via ERK1/2 may be a critical pathway leading to glomerular pathology.

Additionally, there is emerging evidence that the pro-inflammatory transcription factor, NF-κB, plays a key role in the pathogenesis of renal inflammation including GN (Sanz et al., 2010; Song et al., 2019; Lin and Hu, 2022). NF-κB activation and subsequent transcription of multiple proinflammatory genes has been reported in glomerular podocytes, mesangial, tubular, and endothelial cells in acute renal injury or upon exposure to inflammatory stimuli *in vivo* and *in vitro* (Guijarro and Egido, 2001; Sanz et al., 2010; Song et al., 2019). Reports also suggest that NF-κB is a downstream effector of both Ang II/Ang II- type 1 (AT1) receptor signalling axis as well as EGFR signalling and that EGFR inhibitors can attenuate NF-κB signalling as well as the associated inflammation (Mezzano et al., 2003; Akhtar et al., 2015; Benter et al., 2015; Cong et al., 2022). Thus, it is tempting to speculate that Ang II-mediated shedding of EGF-like ligands, subsequent transactivation of EGFR and activation of the pro-inflammatory NF-κB could be a likely mechanism in the development of GN–though this requires further study and verification *in vivo*.

In summary, the above studies provide emerging evidence for upregulated EGFR signalling, either via direct ligand-mediated activation or via Ang-II-mediated transactivation, and the possible involvement of downstream effectors, such as ERK1/2 and/or NF-κB, to be critical in the development of GN (for a summary see also Table 1).

## 3 Diabetic nephropathy

Diabetic nephropathy (DN) is the leading cause of end-stage renal failure worldwide (Duran-Salgado and Rubio-Guerra, 2014; Li et al., 2022; Nomura et al., 2022). In particular, one-third of all type 1 diabetes (T1D) patients along with around 25% of type 2 diabetic (T2D) patients are affected (Duran-Salgado and Rubio-Guerra, 2014). Chronic or prolonged intermittent hyperglycemia associated with diabetes mellitus can lead to a general dysfunction in the kidney (e.g., in mesangial cells, podocytes, endothelial and tubular epithelial cells) through multiple pathological mechanisms including excess production of reactive oxidative species (ROS) or reactive nitrogen species (RNS), and downstream activation of several signaling networks including EGFR (for a recent review, see also Sheng et al., 2021). DN arises from the subsequent dysfunction in renal cell growth/proliferation, angiogenesis, and/or apoptosis and clinically manifests with symptoms of proteinuria together with morphological changes or remodeling in the glomerulus and interstitium (Sheng et al., 2021). Its complex pathogenesis may also involve low grade inflammation as well as hemodynamic and metabolic disturbances (Sheng et al., 2021).

Several reports have now shown that renal EGFRs are involved in DN (for a summary, see Table 2). Upregulation of EGFR, and other ErbBs, has been reported in conditions of hyperglycemia and/or diabetes (Konishi and Berk, 2003; Benter et al., 2005a; Benter et al., 2005b; Saad et al., 2005; Portik-Dobos et al., 2006; Uttarwar et al., 2011; Akhtar et al., 2012; Akhtar et al., 2013; Akhtar et al., 2015; Benter et al., 2015; Li et al., 2015; Akhtar et al., 2019). Inhibiting EGFR or its downstream signaling is protective against the development of DN and as such might be exploited as a potential new therapeutic strategy in its treatment. In a comprehensive study, Li et al. tested if blocking the activation of EGFR with Erlotinib could influence the development of DN in an accelerated T2D mouse model (Li et al., 2018b). Compared to controls, the Erlotinib-treated eNOS deficient db/db mice had markedly decreased albuminuria, glomerulosclerosis, podocyte loss, and fibrosis (Li et al., 2018a). In addition, the intervention reduced the infiltration of the immune cells such as the macrophages and T-lymphocytes, decreased the renal oxidative stress, and macrophage and T-lymphocyte infiltration, as well as fewer proinflammatory cytokine levels. Interestingly, Erlotinib also exerted anti-diabetic actions in this mouse model. Thus, apart from the kidneys, EGFR inhibition was also reported to preserve pancreatic function and enhance insulin levels in eNOS deficient *db/db* mice. Erlotinib also decreased fasting blood glucose levels along with increased glucose tolerance, insulin sensitivity and circulating levels of the adipokine, adiponectin-that has reno-protective and insulin sensitizing actions (Li et al., 2018b). Thus, this dual action upon EGFR inhibition might be particularly useful in DN. However, it should be noted that EGFR inhibition or deletion does not always lead to a lowering of blood glucose levels as observed in several other animal models (Benter et al., 2005a; Benter et al., 2005b; Akhtar et al., 2013; 2019; Benter et al., 2015; Xu et al., 2017; Li et al., 2021) despite a couple of case studies in human lung cancer patients have pointed to anti-diabetic effects with Erlotinib (Costa and Huberman, 2006; Brooks, 2012). The potentially beneficial action of EGFR inhibitors in diabetes, and specifically in DN, requires further confirmation and validation in large scale clinical studies in human subjects.

**TABLE 2:** Summary of selected studies highlighting the role of EGFR signalling in diabetic nephropathy.

Number	Model	Methodology	Findings/Results/Conclusion relevant to EGFR	Study Reference
1	T2D mice model	Vehicle or Erlotinib at 8 weeks of age up to 20 weeks	Erlotinib reduced, kidney damage, oxidative stress, immune cell infiltration, maintained pancreatic function, and increased adiponectin	Li et al. (2018b)
2	WT vs. mutated endothelial nitric oxide synthase mice	5 days injections of STZ followed by daily Erlotinib forced feeding	Erlotinib prevented DN in T1D by inhibiting mTOR and activating AMPK, with augmented autophagy and inhibition of ER stress	Zhang et al. (2014)
3	Diabetic mice treated with EGFR inhibitor	Diabetes mellitus was induced, followed by EGFR inhibition	EGFR/AKT/ROS/ER stress-induced pathways have a crucial role in the development of DN	Xu, Z et al. (2017)
4	Mesangial cells	Pharmacologic inhibitors followed by either glucose or mannitol	Hyperglycemia results in EGFR activation which controls matrix upregulation in mesangial cells	Uttarwar et al. (2011)
5	WT vs. podocyte-specific EGFR knockout mice	Both models had induction of diabetes using streptozotocin injection	Upregulation of EGFR has a crucial role in the injury and loss of podocytes in DN	Chen, J et al. (2015), Rintala et al. (2016)
6	Human proximal tubular cells	High glucose with or without pioglitazone followed by gefitinib	EGFR inhibition along with PPAR-γ agonist lessens hyperglycemia-induced fibrosis and inflammation. It also prevents the retention of sodium and water in the proximal tubular cells	Pegg et al. (2013)
7	Mice and human podocytes	Cells were placed in collagen-coated dishes to induce differentiation	Activation of EGFR causes ZO-1 redistribution which plays a crucial role in BK-induced change in permeability	Dey et al. (2010)
8	Sprague-Dawley primary rat MC	High glucose or mannitol	Upregulation of ADAM17 is linked to a stronger activation and a strengthened effect on matrix production	Li, R et al. (2015)
9	Primary rat mesangial cells	Transfection with ADAM17 when indicated	Findings suggest a crucial function of FAK in ADAM17 activation	Li et al. (2018a)
10	proximal tubule-like epithelial cells	PCT EGFR deletion mice or Erlotinib administration	Hippo pathway is linked to renal epithelial injury mediated by EGFR	Chen and Harris (2016)
11	Rat proximal tubule cells	One-month vorinostat treatment	Epigenetic intervention can prevent acute renal changes in diabetes	Gilbert et al. (2011)
12	Mice model	Vehicle, insulin, or recombinant human (rh)NRG-1	rhNRG-1 in hypercholesterolemic T1D diabetic mice prevented cardiovascular and renal complications	Vandekerckhove et al. (2016)
13	Human mesangial cells, and mice models	Measuring renal TGFA mRNA expression in human DKD	The findings suggest a pathologic contribution of TGFA in DKD	Heuer et al. (2017)
14	Mice model	Studying the role of EGFR in DN	PTHrP upregulates fibronectin in mesangial cells	Chen et al. (2019b)
15	p47phox null mice	Adriamycin or partial renal ablation	NADPH oxidases dependent on p47phox play a role in kidney injury through ROS	Wang et al. (2015)
16	Gprc5a mutant mouse	Molecular studying of GPCRs	Gprc5a is podocyte-specific and was markedly reduced in models with DN	Ma et al. (2018)
17	Mice model	Measuring the expression of miR-146a in T2D	The decreased expression in T2D is linked with kidney damage	Lee, HW et al. (2017)
18	Rats’ mesangial cells	Investigation of SREBP-1 activation via hyperglycemia	Activation of SREBP-1 results in upregulation of TGF-β1	Uttarwar et al. (2012)
19	Human proximal tubular epithelial cells	Measuring the levels of ARAP1 and ARAP1-AS2 in human proximal tubular epithelial cells	EGFR-specific pathway activation by lncRNA ARAP1-AS2 is seen due to hyperglycemia which induces proximal tubular cells injury	Li, X et al. (2020)

In another study, Zhang et al. (2014) showed beneficial effects of Erlotinib treatment on the progression of DN in mouse models of T1D through attenuation of the albumin/creatinine ratio, reduced histological glomerular injury, reduced renal expression of connective tissue growth factor, and collagens I and IV. Furthermore, mice treated with Erlotinib had indications of enhanced renal autophagy which reduces the endoplasmic reticulum (ER) stress and thus renal tissue injury. Furthermore, treated mice also had proof of AMP-activated protein kinase activation which inhibits the mammalian target of rapamycin (mTOR) pathway, an important factor in the development of DN and an inhibitor of autophagy (Zhang et al., 2014). These findings were further supported by Xu et al. (2017) who showed that oxidative stress and ER stress were ameliorated following treatment with AG1478, a specific inhibitor of EGFR phosphorylation, in the same mouse model. In addition, pro-fibrotic genes such as TGF-β and collagen IV were shown to be decreased by AG1478. The intervention also reduced oxidative and ER stress that results from high glucose levels in renal mesangial SV40 cells. The results showed that EGFR/AKT/ROS/ER stress signaling is crucial in the development of DN (Xu et al., 2017).

Several mechanistic studies have aimed to provide insight into the likely underlying mechanisms by which activation of ErbBs and/or shedding of their ligands might lead to DN *in vitro* and *in vivo*. Indeed, several of the EGF-like ligands that can activate multiple members of the EGFR family of receptors are now thought to be involved in the pathogenesis of DN. For example, in mesangial cells exposed to hyperglycemia, general inhibition of matrix metalloproteases, MMPs, (known to be involved in EGF-like ligand shedding) or specifically of HB-EGF ligand inhibited the activation of both, EGFR and downstream AKT signaling (Uttarwar et al., 2011; Uttarwar et al., 2012). Another ligand that plays a role in multiple kidney pathologies, including DN, is TGF-α. Antibody-mediated blockade of TGF-α reduced prolonged EGFR signaling in renal tubular and mesangial cells (Heuer et al., 2017). This study implicated TGF-α as a pharmacological target that can play a role in treating kidney diseases. Thus, it appears that targeting the upstream mediators of EGFR transactivation (e.g., MMP-mediated HB-EGF shedding) or its direct ligands such as TGF-α could also represent a viable therapeutic approach for managing DN.

Another set of enzymes involved in the shedding of EGF-like ligands in the kidney are the family of ADAMs (a disintegrin and metalloproteases). Of these, ADAM 17 has been implicated in several renal pathologies including DN. For example, ADAM17-mediated shedding of epiregulin has been implicated in the beneficial actions of bradykinin (BK), whose levels are typically elevated upon clinical treatment of chronic kidney disease with angiotensin-converting enzyme inhibitors. BK decreases mouse podocyte permeability by acting on the tight junction protein zonula occludens-1 (ZO-1) (Dey et al., 2010). These actions of BK2 receptor appear to involve ADAM-17-dependent EGFR transactivation. By inhibiting the EGFR receptor, the ZO-1 rearrangement and BK-induced decrease in the permeability of podocytes was attenuated (Dey et al., 2010). In addition, ADAM17 mediates hyperglycemia-induced matrix production in mesangial cells (Li et al., 2015; Li et al., 2018a). Interestingly, this study showed that ADAM17 upregulation in conditions of hyperglycemia might be self-augmented via an EGFR/ADAM17 signaling loop (Li et al., 2015) and further implicated ADAM17 as a target in treating diabetic kidney disease (Li et al., 2015). In summary, the above studies provide evidence that ADAMs, in addition to EGFR, are potential therapeutic targets in DN and possibly other chronic kidney pathologies.

Interestingly, in contrast to other EGFR ligands discussed above, NRG-1 (neuregulin-1) has been reported to be reno-protective in a model of type 1 diabetes bearing a mild form of nephropathy with significant cardiovascular dysfunction (Vandekerckhove et al., 2016). Recombinant human neuregulin-1 (rhNRG-1) treatment provoked a systemic activation of both ErbB2 and ErbB4 receptors in the model hearts and kidneys. In addition to the multiple cardiovascular benefits, rhNRG-1 significantly reduced albuminuria, neutrophil gelatinase-associated lipocalin, and glomerular fibrosis. This ligand also inhibited the synthesis of collagen of mesangial cells *in vitro*, although it showed no effect on Ang II-induced vasoconstriction of glomerular arterioles (Vandekerckhove et al., 2016). The authors conceded that their model of nephropathy did not mimic the full hallmarks of the disease and thus, for a better understanding of the role of NRGs in DN, it would be necessary to confirm these findings in better models of DN that more closely resemble the characteristics of human DN.

Apart from increased production of EGF-like ligands in DN, it was found that in the glomeruli and tubules of patients with diabetic kidney disease, the parathyroid hormone-related protein (PTHrP) is also upregulated. Additionally, reactive oxygen species derived from nicotinamide adenine dinucleotide phosphate (NADPH) oxidases (NOX) facilitate the activation of PTHrP (1–34)-induced Src kinase which activates the EGFR (Chen et al., 2019a). The subsequent sequelae result in excessive protein synthesis of fibronectin. PTHrP’s role in fibronectin upregulation adds to the efforts dedicated to treating glomerular sclerosis (Chen et al., 2019b). The main generators of reactive oxygen species (ROS) in the glomerulus (P47phox, NOX1, and NOX2) have now been implicated in the pathogenesis of many kidney diseases including DN. P47phox, that regulates the formation as well as the function of NOX1 and NOX2, has been shown to exacerbate DN (Sheng et al., 2021). Indeed, P47phox deletion safeguarded mice from developing glomerulosclerosis and albuminuria (Wang et al., 2015). These results showed that P47phox and its dependent NADPH oxidases could be potential targets to attenuate fibrotic disease through antioxidant therapy (Wang et al., 2015). Oxidative stress pathways were also implicated in a study on podocyte-specific EGFR knockout diabetic mice which exhibited markedly decreased albuminuria and podocyte loss when compared to their wild-type littermates (Chen et al., 2015). The study also showed that podocyte-specific EGFR knockout mice had reduced TGF-β1 expression, Smad2/3 phosphorylation, and glomerular fibronectin deposition. The alterations seen in the wild-type mice were attenuated by treatment with antioxidants or by preventing EGFR expression or activity. Therefore, it is likely that NOX-mediated EGFR signaling is essential in the downstream activation of pathways that facilitate podocyte injury and loss in DN (Chen et al., 2015).

A more recent study by Li et al. 2021 showed that selective podocyte EGFR deletion led to relative podocyte preservation and marked reduction in albuminuria and glomerulosclerosis, renal proinflammatory cytokine/chemokine expression, and decreased profibrotic and fibrotic components in nos3 −/−; db/db mice. Podocyte EGFR deletion led to decreased podocyte expression of the autophagy-regulator, rubicon ((RUN domain and cysteine-rich domain containing Beclin one-interacting protein) and was associated with increased podocyte autophagy activity. Therefore, activation of EGFR signaling in podocytes contributes to progression of DN at least in part by increasing rubicon expression resulting in subsequent attenuation of autophagy and podocyte injury.

Pegg et al. (2013) provided further evidence which demonstrated that using EGFR inhibitor with a peroxisome proliferator-activated receptor gamma (PPAR-γ) agonist attenuates the fibrosis and inflammation resulting from hyperglycemia. In addition, it also counteracts the activation of the channels and transporters that play a role in the retention of sodium and water in human proximal tubular cells. These data shows that blocking EGFR, apart from limiting the tubulointerstitial pathology in DN, also reduces the retention of sodium and water noted in diabetics. This effect is further induced by the PPAR-γ activators. (Pegg et al., 2013).

A novel mechanism was postulated by Chen and Harris (2016), who showed that upregulation of Yes-associated protein (YAP) that is seen in diabetic patients which is EGFR-dependent is crucial in the upregulation and expression of the profibrotic factor CTGF and amphiregulin. For the first time, these findings demonstrated the interaction between EGFR signaling and the Hippo pathway which could be a crucial step in the pathogenesis of DN (Chen and Harris, 2016). Thus, further studies are necessary to evaluate whether targeting of the Hippo pathway alone or in combination with EGFR signaling could more effectively slow the progression of DN in patients.

The acetylation status of histone proteins can alter EGF-EGFR signaling in renal tubule cells. Gilbert et al. evaluated the effects of vorinostat, a histone deacetylase (HDAC) inhibitor, in attenuating the enlargement of the kidneys in diabetics with a focus on the EGF interaction with EGFR (Gilbert et al., 2011). In diabetic mice treated with vorinostat, the results showed a reduction in the proliferation of the tubular epithelial cells. Treatment with vorinostat daily for a month reduced the glomerular hypertrophy and renal growth, which the authors claimed was most likely due to downregulation of EGFR following blockade of epigentic changes in histone acetylation (Gilbert et al., 2011). These data suggest that modulation of epigenetic changes might prevent early renal changes in DN.

Several other therapeutic targets have been proposed for diabetic nephropathy. Among these is a novel molecular target, Gprc5a, which is only located within the glomerular podocytes and plays an essential role in healthy and diseased kidneys. In a link to EGFR, the stable overexpression of Gprc5a in glomerular podocytes was proven to inhibit EGFR activation (Ma et al., 2018). In addition to other effects like blunting the downstream signaling to the TGF-β pathway, it was concluded that this receptor can serve as a key therapeutic target in treating DN. However, this could be challenging as the drugability assessment showed several challenges that require dedicated investigation (Ma et al., 2018).

MicroRNAs (miRNAs), small non-coding RNAs that control gene expression by regulating target messenger RNA translation, have been implicated in several kidney diseases (Mahtal et al., 2022). As miRNAs are critical regulators of cellular homeostasis, their dysregulation is a crucial component of cell and organ injury (Mahtal et al., 2022). Of particular interest is microRNA-146a (miR-146a) that may regulate expression of ErbBs. The expression of this miRNA is increased in different cells in homeostatic conditions as it has a crucial anti-inflammatory role in myeloid cells. A study showed that the expression of this molecule in the glomeruli is decreased in patients with type 2 diabetes, which is linked with an increase in albuminuria and glomerular damage (Lee et al., 2017). miR-146a deficient mice were found to have faster progression of glomerular disease and albuminuria following induction of hyperglycemia using streptozotocin. In addition, the targets of miR-146a, Notch-1, and ErbB4 were found to be highly upregulated in the glomeruli of diabetic mice and patients (Lee et al., 2017). Consequently, it was shown that using an inhibitor of all ErbB kinase receptors significantly reduced the diabetic glomerular injury and albuminuria in wild-type as well as miR-146a deficient animal models (Lee et al., 2017). Also at the genetic level, ARAP1 is a gene associated with an increased risk for type 2 diabetes. This gene controls the internalization and ubiquitination of membrane receptors. In a recently published study, the antisense long non-coding RNA of this gene (ARAP1-AS2) and the gene itself were investigated to see their effect on the ubiquitination of EGFR in DN (Li et al., 2020). The results of the study showed that ARAP1-AS2 has the ability to interact with ARAP1. This interaction plays a crucial part in the hyperglycemia-induced injury and fibrosis of the proximal tubules by retaining chronic activation of the EGFR/TGF-β/Smad3 pathway (Li et al., 2020). The results showed that ARAP-AS2 and ARAP1 could be potential targets to treat DN.

Another transcription factor that was proven to have a role in DN is the sterol-responsive element-binding protein (SREBP)-1. A study showed that EGFR/PI3K/RhoA signaling is essential for hyperglycemia-induced SREBP-1 activation which then results in TGF- β1 upregulation (Uttarwar et al., 2012). The findings provided further evidence to support the importance of EGFR in DN and presented SREBP-1 as a potential target in treating DN (Uttarwar et al., 2011; Uttarwar et al., 2012).

Finally, diabetes-induced vascular remodeling and hemodynamic changes are also thought to contribute significantly to the development of renal pathologies including DN (Sheng et al., 2021). Chronic hyperglycemia-induced glomerular hyperfiltration arises from asymmetric alterations in arteriolar resistance with greater dilation of the efferent *versus* afferent arterioles that ultimately leads to increased transcapillary hydrostatic pressure (Sheng et al., 2021). This eventually leads to albuminuria. Many molecules, in particular the RAAS octapeptide, Ang Ⅱ, have been implicated in causing hyperfiltration/hyperperfusion. (Cooper, 2001; Wolf, 2004; Forbes et al., 2007; Sheng et al., 2021). Since Ang II mediates transactivation of EGFR, it has been suggested that inhibition of EGFR can reduce Ang Ⅱ-mediated hemodynamic alterations (Krishna et al., 2007; Akhtar et al., 2012; Shraim et al., 2021).

There is a growing body of evidence to suggest that EGFR and related family members are key mediators of diabetes-induced vascular dysfunction (for a recent review see Shraim et al., 2021). Indeed, several *in vivo* studies have now shown that treatment with EGFR inhibitors, mostly without correcting blood sugar levels, can markedly normalize the altered vasoconstrictor and vasodilator responses typically observed in the diabetic vasculature including in the renal artery (Benter et al., 2005a; Benter et al., 2005b; Yousif et al., 2005; Benter et al., 2009a; Akhtar et al., 2012; Akhtar et al., 2013; Schreier et al., 2014; Akhtar et al., 2019). Mechanistically, upregulation of EGFR signaling appears to be a key early vascular change induced by diabetes as EGFR inhibition was able to correct more that 90% of all diabetes-induced gene expressions in the diabetic vasculature (Benter et al., 2009b). Hyperglycemia-induced upregulation of EGFR receptors likely includes other family members especially ErbB2 (Akhtar et al., 2013; Akhtar et al., 2015; Benter et al., 2015). Use of selective inhibitors of EGFR and/other ErbBs were able to reverse multiple diabetes-induced signaling changes including oxidative stress signaling, inflammatory markers, nitric oxide (NO) synthase (eNOS) activity and NO production in the vasculature (Benter et al., 2015; Kassan et al., 2015). In addition to EGFR/ErbBs mediating diabetes-induced vascular dysfunction, EGFR has also been directly linked to diabetes-induced vascular remodeling (Akhtar et al., 2019). Chronic EGFR inhibition resulted in a significant reduction in diabetes-induced blood vessel intima and media thickening and correction in vascular hyper-responsiveness through EGFR-ERK1/2-ROCK dependent pathway (Akhtar et al., 2019). Thus, therapeutic targeting of one or more EGFR/ErbB receptors might be useful in correcting diabetes-induced renal vascular dysfunction as well as for reversing the underlying morphological changes and alterations in key renal cell signaling cascades typically associated with DN.

In summary, the above studies suggest that a complex cascade of signaling networks involving multiple signaling effectors both upstream and downstream of EGFR appear to be involved in the development of DN through modulation of hemodynamic, inflammatory, fibrosis, and oxidative stress pathways amongst others. Importantly, inhibition of EGFR seems sufficient to correct many of the hyperglycemia/diabetes-induced hemodynamic, morphological, and renal cell signaling changes associated with DN.

## 4 Hypertensive nephropathy

After diabetes, hypertension is believed to be the second or third commonest cause of chronic kidney disease as well as renal replacement therapy and poses a significant burden on healthcare globally (Carriazo et al., 2020; Costantino et al., 2021; Wang et al., 2024). Despite the strong association of hypertension with nephropathy, hypertensive nephropathy does not have a clear enough definition to allow diagnosis other than by exclusion (Carriazo et al., 2020). In the absence of uniform criteria to accurately diagnose hypertensive nephropathy, the prevalence of chronic kidney disease resulting from hypertension is therefore difficult to quantify with any precision (Udani et al., 2011; Carriazo et al., 2020). Thus, there appears to be an important need to come up with a clear definition of hypertensive nephropathy with a comprehensive set of criteria to be able to exclude other nephropathies (Carriazo et al., 2020).

The etiology of hypertensive nephropathy appears complex, but key pathological features involve arteriolar nephrosclerosis, a vascular disease characterized by progressive intimal thickening of small arterioles due to persistent elevation in blood pressure, followed by renal parenchymal damage and ischemic/hypoxia-mediated glomerulosclerosis (Freedman and Cohen, 2016; Costantino et al., 2021). Renal microvasculature dysfunction and the associated local ischemia/hypoxia culminates in signaling changes leading to renal inflammation and fibrosis. Additionally, chronic elevated systemic blood pressure causes renal tubular cell injury as well as podocyte effacement and loss, leading to disruption of the renal filtration barrier and microalbuminuria (Costantino et al., 2021), though clinical symptoms often appear much later than the underlying cell signaling and histopathological changes (Sun, 2019; Kao and Huang, 2021).

Several key signaling effectors including RAAS peptides may be involved (Sun, 2019), but recent evidence implicates EGFR as a key player in the pathology of hypertensive nephropathy (Benter et al., 2009a; Tang et al., 2013b; Staruschenko et al., 2013) ((for a summary of selected studies see also Table 3)). We and others have shown that in animal models of hypertension, expression of EGFR is elevated in kidneys (François et al., 2004; Benter et al., 2009a) including in the renal vasculature, such as the media of afferent and efferent renal arterioles (Swaminathan et al., 1996; Ying and Sanders, 2005). In deoxycortisone acetate (DOCA) salt-induced hypertensive mice, early treatment with a selective inhibitor of EGFR was able to prevent changes in more than 97% of the approximately 2,400 genes upregulated in the hypertensive kidney (Benter et al., 2009a). EGFR inhibition with AG1478 concomitantly reduced proteinuria, blood pressure, and histological changes indicative of kidney damage in this mouse model, (Benter et al., 2009a), implying that EGFR is a key early initiator step in the development of hypertension-induced renal dysfunction in this model. Further, it suggested that many of the upregulated genes associated with DOCA–salt hypertensive kidneys are likely downstream of EGFR signaling and thus, highlighting this important receptor tyrosine kinase as a potentially viable pharmacological drug target. In support of this notion, gefitinib -a clinically used EGFR inhibitor-was also reported to counter the progression of glomerular and vascular fibrosis in this model. In addition, inhibition of EGFR also reduced vasoconstriction and improved blood pressure in models of hypertension (Benter et al., 2009a; Tang et al., 2013b). It is known that EGFR expression is elevated in the vascular smooth muscle cells from hypertensive rats and in atherosclerotic plaques that may have an impact on hypertension (Rayego-Mateos et al., 2018b). Thus, further studies are needed to ascertain the relative contribution of EGFR in mediating hypertension *versus* its direct effects on kidney damage *per se*.

**TABLE 3:** Summary of selected studies highlighting the role of EGFR signalling in hypertensive nephropathy.

Number	Model	Methodology	Findings/Results/Conclusion relevant to EGFR	Study Reference
1	Deoxycorticosterone acetate salt hypertensive mice	AG1478 and gefitinib EGFR inhibition	EGFR inhibition prevents hypertensive kidney damage and improves renal function	Tang et al. (2013b)
2	DOCA-salt-induced hypertensive Wistar rats	Micro-array based gene expression analyses of rat kidneys treated with or without AG1478, a selective EGFR inhibitor. Kidney damage assessed by analysis of proteinuria, renal artery responsiveness and histopathology studies	EGFR inhibition prevented upregulation of more than 97% of genes associated with hypertension in the rat kidney and also normalized hypertension-induced proteinuria, renal artery responsiveness and histopathology changes	Benter et al. (2009a)
3	Dahl/Rapp salt-sensitive (S) rats and Sprague-Dawley (SD) rats	Immunohistochemistry using polyclonal antibodies to EGFR	CKD in S is related to arterial pathology following the onset of hypertension. The studies showed augmented expression of EGFR in S rats	Ying and Sanders (2005)
4	Spontaneously hypertensive rats	EGFR expression assessment in mice with hypertension and atherosclerosis	Blocking EGFR attenuated the elevation in BP and reduced vascular injury. Moreover, EGFR was described in atherogenic processes of atherosclerosis	Rayego-Mateos et al. (2018a)
5	Hypertensive chronic kidney disease rats	EGFR inhibition using PKI-166	The treatment prevented worsening of hypertension and preserved cardiac function like lisinopril	Ulu et al. (2013)
6	Peptide array chips in homozygous Ren2 rats	Protein kinase profiling in renal lysates from a rat model of fibrosis treated with or without ramipril, an ACE inhibitor for 4 weeks	ACE inhibition reduced EGFR kinase activity	De Borst et al. (2007)

## 5 Acute kidney injury

Acute kidney injury (AKI) is the abrupt reduction in kidney function in a few hours, which includes structural injury and functional impairment (Makris and Spanou, 2016; Nourie et al., 2024). Even though there is scarcity of data, it is believed that the prevalence of AKI goes up to a little over 13 million cases per year (Ponce and Balbi, 2016). The prevalence is estimated to range from <1% to 66%, where this variability is explained by the inconsistent use of standardized AKI classification criteria (Hoste et al., 2018). AKI can develop in different clinical situations including sepsis, ischemia, and nephrotoxicity. Animal studies of AKI have illustrated that following an AKI, the kidney has the capacity of recovering from the insult. The regeneration process includes the dedifferentiation, proliferation, and migration and ends with redifferentiation into mature tubular cells. It is thought that this regeneration process occurs through the affected renal tubular epithelial cells (RTECs) undergoing differentiation to a mesenchymal stage by reactivating pathways which are common to the early development of the kidneys (Cirio et al., 2014). Different cellular molecules like vimentin and neural cell adhesion molecule 1 (NCAM1) which are no longer expressed after the metanephric mesenchyme stage and kidney maturation are reexpressed during the recovery phase in renal tubular cells. Other molecules such as Pax-2 are markedly expressed in the surviving tubular epithelium although it is not found in adult kidneys. This indicates the presence of cells with immature characteristics. Similarly, another marker of dedifferentiation, Sox9 was elevated up to 30 days following an injury. Moreover, nestin, which is an intermediate filament was proposed as an indicator of dedifferentiation (Andrianova et al., 2019). These findings suggest that renal regeneration and recovery after acute injury share lots of similarities with the early development of the kidneys. During development, dedifferentiation, high proliferation, and regulation by different growth factors such as the EGFR ligands are noted on the renal mesenchymal cells. Experiments showed that the EGFR ligands EGF, TGF-α, epiregulin, and HB-EGF, are upregulated in metanephric structures and play a role in tubulogenesis and breaching (Zeng et al., 2009). Thus, EGFR signaling is thought to be a major regulator of renal development, the proliferative capability of mature proximal tubule cells and in mediating recovery from acute kidney injury (for a summary of selected studies see also Table 4). Increased phosphorylation of the EGFR was noted in the proximal tubular cells in different experimental models of AKI, including hypoperfusion/reperfusion, aminoglycoside toxicity, and administration of folic acid (He et al., 2013; Gao et al., 2020). Further, EGFR/FOXM1 signaling is known to induce proliferative repair after kidney injury (Chang-Panesso et al., 2019).

**TABLE 4:** Summary of selected studies highlighting the role of EGFR signalling in acute kidney injury (AKI).

Number	Model	Methodology	Findings/Results/Conclusion relevant to EGFR	Study Reference
1	Kim1-GCE mice and Cell Lineage	Determining the cellular transcriptome of proximal tubule during repair in acute ischemic kidney injury	Repair is driven by EGFR/FOXM1-dependent signaling pathways	Chang-Panesso et al. (2019)
2	AKI Clinical patients RPTC and knockout Mice (deletions of Yap and Taz)	Hypoxia-reoxygenation of cultured human RPTCs and knock-out mice subjected to bilateral IRI	Activation of EGFR-Dependent YAP (Yes-associated protein) is necessary for renal repair from Acute Kidney Injury	Chen, J et al. (2018)
3	Murine	AKI in Waved −2 and Gefitinib treated mice, induced by folic acid	Genetically and pharmacologically EGFR impaired activation mice had increased renal dysfunction	He et al. (2013)
4	Murine	Inhibiting ERK1/2 activation by Erlotinib	Attenuation of the early reduction in PGC-1α along with prevention of diminishing kidney function	Collier et al. (2016)
5	*In vivo* zebrafish	Sec10 overexpression in renal tubular MDCK cell and knockdown of sec10 in vivo in zebrafish	Increased susceptibility of renal tubule cells to damage when EGFR, MAPK, and endocytosis-dependent pathways are inactivated	Fogelgren et al. (2014)
6	LPS-induced renal injury mouse	Pharmacological inhibitors of EGFR effect on Hexokinase activated due to renal injury	Renal cortical HK activity enhanced in LPS model via EGFR- and Akt	Smith et al. (2014)
7	Mouse	Class I HDACs inhibition with MS-275	EGFR phosphorylation halted and diminished its expression, resulting in more severe tubular injury	Tang et al. (2014)
8	C57BL/6 mice	Ischemic AKI mice were subjected to bilateral renal IRI. In toxic AKI model, mice were exposed to a single cisplatin intraperitoneal injection	MMP-10 defends against AKI by enhancing EGFR signaling	Hu et al. (2021)

While growing evidence shows that renal recovery from AKI is a result of dedifferentiation and proliferation of surviving tubular epithelial cells, two pathways seem to have a crucial role in the proliferation and differentiation process, namely, the EGF receptor (EGFR) and the Hippo signaling pathway (Harris, 2021). Several articles have proven that the activation of EGFR in renal proximal tubule epithelial cells (RPTCs) had a major role in preventing ischemia-reperfusion injury (IRI). It was proven that EGFR-Dependent YAP (Yes-associated protein) upregulation is necessary for renal recovery from AKI (Chen, J et al., 2018). These results come in line with another study that supported the evidence that activation of EGFR is critical for the onset of dedifferentiation and proliferation of the renal tubular cells after inducing acute kidney injury by folic acid (He et al., 2013). This was further supported by another model which provided genetic and pharmacologic proof that activation of EGFR in the proximal tubular cells is crucial in the recovery from AKI, by generating mice with EGFR mutation in RPTCs (Chen et al., 2012). Ischemia-reperfusion induced AKI resulted in significant activation of EGFR in control mice, unlike the genetically mutated (i.e., knockout) mice or the wild-type which were treated by Erlotinib which had no functional EGFR. It was noted that kidney injury was reduced in the control mice compared to the knockout and Erlotinib-treated mice which had chronic dilation of the proximal tubules, epithelial simplification, as well as cast formation. In addition, the proliferation of the renal cells was slowed because of decreased ERK and AKT signaling (Chen et al., 2012).

Regarding the precise signaling mechanisms in the kidney, phosphorylation of EGFR was noted as an upstream activator of ERK1/2 (extracellular signal-regulated kinase), through negative regulation of PGC-1α transcription in normal physiological conditions. However, under the abnormal/pathological state of renal ischemia/reperfusion-induced AKI in mice, the activation of ERK1/2 resulted in a decrease in PGC-1α transcription and a decline in kidney function as indicated by serum creatinine level. Through inhibition of EGFR-ERK1/2, Erlinotib treatment halted the initial reduction in PGC-1α and stopped the decline in kidney function (Collier et al., 2016). Furthermore, a study in renal tubular MDCK cells showed that the mechanism by which the overexpression of exocyst Sec10, which is a highly conserved eight-protein complex that regulates protein trafficking, increases phosphorylation of ERK and healing from oxidative injury is EGFR, MAPK, and endocytosis dependent (Fogelgren et al., 2014), thereby highlighting the potential use of exocyst Sec10 as a therapeutic target in AKI therapy.

Many previous reports have indicated that glycolysis provides an alternative way of providing energy following tubular injury, which under normal conditions, uses oxidative phosphorylation for ATP generation with low glycolytic capacity. To investigate if glycolysis occurs in AKI, LPS-induced renal injury in a mouse model indicated a particular acute increase in hexokinase (HK) function. This activation was halted by pharmacological inhibition of EGFR, showing that LPS increases HK function in the renal cortex occurs via EGFR-dependent mechanisms (Smith et al., 2014).

Although the fact that histone deacetylases (HDACs) activation is crucial for the proliferation of renal epithelial cells and the development of the kidney, it remained unclear whether they play a role following an AKI. To shed light on their significance, a study inhibited class I HDACs which showed suppression of EGFR phosphorylation as well as a reduction in its expression, suggesting that this class protects the kidney, helps in regaining kidney function and is necessary for renal regeneration after acute kidney injury (Tang et al., 2014).

Matrix metalloproteinase-10 is a zinc-dependent endopeptidase that plays a role in various biological processes. It is upregulated in the tubular epithelium in models of AKI provoked by ischemia, reperfusion, or pharmacologically through cisplatin. The increased expression of exogenous MMP-10 improved AKI, while the knockdown of endogenous MMP-10 worsened AKT. Both findings resulted from activating and inhibiting EGFR signaling pathways respectively, that modulated the downstream AKT and extracellular signal-regulated kinase-1 and 2 (ERK1/2) signaling (Hu et al., 2021).

Another molecule is Lysophosphatidic acid (LPA) which raises the production of platelet-derived growth factor-B (PDGFB) and connective tissue growth factor (CTGF) by proximal tubular (PT) cells via LPA2 receptor-Gqα-αvβ6- activation of transforming growth factor-β1 (TGFB1) mediated by integrin. These secretions increase after ischemia-reperfusion injury to the kidneys, paving the way for kidney fibrosis (Geng et al., 2021). Recent findings prove that LPA1 receptor signaling via EGFR-ERK1/2-activator protein-1 conjoins with LPA2-dependent TGFB1 signaling and increases PDGFB/CTGF formation and secretion by PT cells (Geng et al., 2021). Therefore, it is proposed that antagonists of the TGFB1 receptor, LPA1, and LPA2 inhibitors can be used in AKI to prevent the development of CKD (Geng et al., 2021) (see also Table 4).

In summary, consistent with its ‘Jekyl and Hyde’ role, EGFR signaling network is needed for recovery and repair of kidneys following AKI but prolonged EGFR activation can lead to unwanted pathologies such as renal fibrogenesis post-AKI (Tang et al., 2013a; Tang et al., 2013b).

## 6 Chronic kidney disease and its progression from AKI

Chronic kidney disease (CKD) is a description of many different diseases that disturb the kidneys’ structure and function (Levey and Coresh, 2012; Yeh et al., 2024). CKD is diagnosed when there is evidence of kidney damage or reduced function based on the glomerular filtration rate (below 60 mL/min) for at least 3 months, regardless of the clinical diagnosis. The incidence of CKD goes up to 200 per million per year in many countries. The average survival in the USA is 3–5 years, and the prevalence is approximately 1800 cases per million. The higher prevalence is noted in countries with high survival like Japan and Taiwan, with around 2,400 cases per million. CKD is most commonly caused by diabetes, and the disease has some genetic and environmental factors that contribute to its development (Levey and Coresh, 2012).

Multiple research articles have linked EGFR with CKD (for a summary of selected studies see also Table 5). Activation of EGFR results in several cellular responses that depend on the ligand that is bound to it. A major ligand of interest in relevance to CKD is CTGF/CCN2 which when bound to EGFR controls renal inflammation, cellular growth, and fibrosis. In addition, other ligands are also linked to kidney disease, including Ang II, TGF-β, and cytokines, including members of the TNF superfamily (Rayego-Mateos et al., 2018b). Regarding the therapeutic approach, CTGF is being considered as a target because of its pro-inflammatory and fibrotic properties, however, further evidence is required to recognize the consequence of its binding to EGFR. Nevertheless, it is proven that EGFR and some of its ligands could be targeted in treating CKD (Rayego-Mateos et al., 2018b).

**TABLE 5:** Summary of selected studies highlighting the role of EGFR signalling in Chronic Kidney Disease.

Number	Model	Methodology	Findings/Results/Conclusion relevant to EGFR	Study Reference
1	Review Paper	EGFR activation by various ligands, in different tissues and pathologies	CTGF/CCN2 binds with EGFR and results in the regulation of kidney inflammation, cell growth, and fibrosis	Rayego-Mateos et al. (2018b)
2	Human proximal tubular epithelial cells and NRK-49F rat renal fibroblasts	Rac enhances tubulointerstitial fibrosis and (CKD)	Rac-GTPase enhances fibrotic TGF- β1 signaling as well as CKD through multiple pathways including EGFR	Patel et al. (2019)
3	Mouse models of LCN2−/− and LCN2^+/+^	Lcn2 function in the regulation of EGFR trafficking	Lcn2 amplifies the cell surface expression of EGFR	Yammine et al. (2019)
4	Injured kidneys *In vitro* proximal tubule cells and primary tubular cells and different mice models	Knockout AREG in proximal tubule cells of either ischemia-reperfusion injury or unilateral ureteral obstruction in mice	Amphiregulin amplifies and plays a main role in profibrotic signals *in vitro* and is needed for fibrosis that resulted from an injury in mice	Kefaloyianni et al. (2019)
5	Different mice models, including ADAM17ex/ex mice	AREG and its profibrotic actions in the mice models	ADAM17 pathway activation in proximal tubules results in activating EGFR and hence fibrosis post kidney injury	Kefaloyianni et al. (2016), Rintala et al. (2016)
6	Review Paper	EGFR activation and its association with renal fibrogenesis post-AKI	Severe AKI results in sustained would lead to fibrogenesis after 28 days	Tang et al. (2013b)
7	EGFR mutant (Wa-2) mice	Halting EGFR using gefitinib and EGFR mutated mice	EGFR deactivation in decreases the complications of vancomycin-induced nephrotoxicity	Xu, L et al. (2019)
8	Rats treated with rat bone marrow = derived mesenchymal stem cells post = AKI	*In vitro* induction of AKI to evaluate miR-146b in renal tubular epithelial cells	Enhanced proliferation of injured renal TECs after inducing ErbB4 post-miR-146b inhibition	Zhu, Y et al. (2016)
9	Renal interstitial fibroblasts of rats and immortalized mice renal PTC	Exposure of fibroblasts to necrotic RPTC supernatant (RPTC-Sup)	Renal fibroblasts were inhibited from substances released by the damaged fibroblasts via dephosphorylation of EGFR by PTP1B activation mechanism	Ponnusamy et al. (2013)

In addition, recent evidence has shown that EGFR activation was also linked to Rac-GTPases which are important regulators of cytoskeletal remodeling (Patel et al., 2019). The deregulation of Rac-GTPases is a main contributor to numerous pathologies. In the context of CKD, Rac-GTPase activates the EGFR, p53, and Hippo/YAZ/TAZ pathways which results in TGF- β1 signaling that promotes fibrosis and CKD. Inhibition of the Rac pathway inhibition was shown to suppress renal free radical damage and maladaptive recovery which made it a new target in treating CKD (Patel et al., 2019).

Another cellular molecule that is linked to EGFR activation and is observed in several pathologies is lipocalin-2 (Lcn2). It was reported that this molecule causes an increased expression of EGFR on the cell surface which is needed for TGF α-induced EGFR salvaging to the plasma membrane and constant activation (Yammine et al., 2019). Lcn2 does this function by inhibiting the lysosomal degradation of the intracellular domain of EGFR. The inhibition of Lcn2 gene activation prevents EGFR recycling and expression in a trial model of CKD. The inhibition resulted in a significant reduction of kidney damage which identifies Lcn2 as a major target to offset the damaging outcomes of EGFR signaling in CKD (Yammine et al., 2019).

Although research has shown that the activity of EGFR is important in the acute phase of recovery in AKI (day 1–2 after injury), the constant activation beyond this phase of days to weeks after injury was found to be associated with kidney fibrosis in experimental models of bilateral ischemic AKI and unilateral ureteral obstruction (UUO). To identify which ligands are responsible for the chronic activation of EGFR and consequent fibrosis, it was found that amphiregulin, a low-affinity ligand for EGFR whose expression is increased following an AKI, drives, amplifies, and integrates profibrotic signals in the proximal tubule cells and is crucial and adequate for AKI-induced fibrosis in mice *in vivo* (Kefaloyianni et al., 2019).

These findings also correlate with another study which reported that ADAM17 pathway upregulation and activation in the proximal tubule results in constant activation of EGFR and fibrosis following different types of kidney injury including ischemia and ureteral obstruction. This occurs through different cellular mechanisms including the EGFR ligand substrate pro-amphiregulin and EGFR-TNFα interaction (Kefaloyianni et al., 2016; Harris, 2021). Similar studies supported this proposal, of which Waved-2 (Wa-2) mice with EGFR reduced activity were compared to normal mice following ischemic renal injury as an exposure. The results showed that severe kidney damage causes prolonged activation of EGFR which is essential for the recovery of renal tubular cells in the initial phase (the first 2 days), but this sustained EGFR activation would lead to fibrogenesis after 28 days (Tang et al., 2013a).

To study the mechanism of the progression of AKI to CKD induced by vancomycin, a model of a mutated EGFR (Wa-2) mice and Gefitinib were used to deactivate EGFR. It was found that AKI caused by vancomycin was significantly reduced in waved-2 mice in the acute phase. While the latter stage ameliorated the renal fibrosis and inflammation. Hence, the study highlighted the significance of EGFR deactivation in decreasing the complications of vancomycin-induced nephrotoxicity (Xu et al., 2019). To further investigate the role of drug-induced AKI, cisplatin-induced acute kidney injury mice treated with rat bone marrow mesenchymal stem cells have been studied. It was found that the expression of microRNA-146b (miR-146b) was increased in AKI rats as opposed to healthy counterparts. In addition, the expression lessened after mesenchymal stem cell therapy. The use of cisplatin *in vitro* augmented the expression of miR-146b renal TECs. This all comes into significance when it was identified that ErbB4 is targeted by miR-146b, its inhibition resulted in an increase in the expression of ErbB4, causing an enhancement in the proliferation of injured renal TECs. Furthermore, this restoration process via rMSCs can be controlled by downregulating ErbB4. Thus, indicating that inhibition of miR-146b is a potential therapeutic option for acute kidney injury (Zhu et al., 2016). In line with this, a study illustrated that products released by injured epithelial cells have the ability to halt renal fibroblasts through dephosphorylation of EGFR by a mechanism involving activation of PTP1B. Given the importance of peritubular fibroblasts in preserving the structural and functional integrity of the kidneys, their inactivation through damage via any insult will impair the process of regeneration of renal epithelium following an AKI (Ponnusamy et al., 2013).

## 7 Renal transplant

Renal transplant is the management option for end-stage renal disease because of its survival benefits in the short and long term compared to dialysis (Kälble et al., 2005; Goto et al., 2024; Jørgensen et al., 2024). The incidence of CKD that is treated by transplantation is limited depending on the difference in the underlying disease rates and the availability of government-sponsored treatment (Levey and Coresh, 2012). The transplanted kidney survival rates are 95% when taken from a living donor and 89% from a deceased donor during the first year after the transplant. A successful renal transplant is influenced by allograft rejection and serious infections (Shams et al., 2017; Sawinski and Blumberg, 2019). Proper preventative measures and prophylaxis are essential to guarantee successful transplantation.

To date, the mechanisms of renal allograft rejection are not fully understood. However, polymorphisms in EGFR may be implicated. For example, Kim et al. 2020 showed that specific single nucleotide polymorphisms (SNPs) displayed a significant association with acute renal allograft rejection in Korean patients. The study involved 347 patients who received their first renal transplant following end-stage renal disease, out of which 63 had acute renal allograft rejection. Among the results, it was found that the SNP (rs11568835) in the epidermal growth factor gene had a significant association with the susceptibility to allograft rejection. In addition, (rs1050171) SNP in the EGFR showed a significant association with susceptibility to allograft rejection as well (Kim et al., 2020).

An additional study looked at the expression of genes using records of allograft biopsies and blood, and it comprised patients experiencing rejection as well as patients with steady kidney function. Based on the biopsy samples, the researchers found out that PIK3R2 and EGFR are the major signaling molecules in inflammatory-immune pathways (Meng et al., 2018). Other studies have proposed the use of TGF-β1, AGT, and EGFR mRNA levels in urine samples of KTP as timely indicators of chronic allograft nephropathy (CAN) development (Mas et al., 2007a; Mas et al., 2007b). However, more research is needed to verify the clinical importance of these findings (See also Table 6 for a summary).

**TABLE 6:** Summary of selected studies highlighting the role of EGFR signalling in Renal transplant.

Pathology	Model	Methodology	Findings/Results/Conclusion relevant to EGFR	Study Reference
Renal Transplant	Patients who received renal transplants following CKD	Genotype analysis of SNP using the Axiom TM genome-wide human assay	SNP (rs11568835) in the EGF gene and SNP (rs1050171) in the EGFR gene had major associations with the vulnerability to allograft rejection	Kim et al. (2020)
Renal Transplant	Blood and allograft biopsies	PIK3R2 and EGFR are the major signaling elements in the pathways involving inflammatory-immune basis established on biopsy samples		Meng et al. (2018)
Renal Transplant	Urine samples of KTP	Possibility of using TGF-β1, AGT, and EGFR mRNA levels in urine. EGFR was downregulated in chronic allograft nephropathy		Mas et al. (2007a), Mas et al. (2007b)

## 8 Renal fibrosis

The common result in end-stage renal disease is renal fibrosis (chronic kidney disease), where renal fibroblasts abnormally activate and grow, along with overproduction of extracellular matrix proteins (Huang et al., 2023; Nørregaard et al., 2023). Around half of the adults above age 70% and 10% of the world’s population develop the outcomes CKD and renal fibrosis, with the incidence increasing worldwide (Huang et al., 2023; Nørregaard et al., 2023). After AKI, tissue repair is able to return the integrity of the damaged tissue only when the injury is mild. However, if the injury was severe, it could lead to renal fibrosis that is known to occur via sustained EGFR activation has been linked to the development of renal fibrosis (Cao et al., 2023; Tang, et al., 2013a; Xu et al., 2019).

Expression of EGFR was elevated in interstitial myofibroblasts in human and mouse fibrotic kidneys and its selective deletion in the fibroblast/pericyte cells inhibited interstitial fibrosis resulting from unilateral ureteral obstruction, ischemia or nephrotoxins (Cao et al., 2023). Additionally, increased HER2 expression in unilateral ureteral obstruction-induced renal fibrosis, type 1 and type 2 diabetic nephropathy, and in kidney biopsies from patients with renal fibrosis has also been reported (Li et al., 2019).

Furthermore, in a mice model study, the progression of chronic kidney disease in rats with remnant kidneys was found to be attenuated using Erlotinib (Yamamoto et al., 2018). An EGFR mimotope (that prevents downstream signaling) alleviated renal fibrosis in the murine unilateral ureteral obstruction model (Yang et al., 2019). Renal fibrosis in Ang II-stimulated mice was also prevented using a new EGFR inhibitor (Skibba et al., 2016). Similarly, supportive of the crucial role that EGFR plays in renal fibrosis induced by Ang II, Ang II-treated SV40 mesangial cells *in vitro* were administered novel small molecules or short hairpin RNA as inhibitors of EGFR, that resulted in preventing the accumulation of connective tissue growth factor (CTGF) and collagen IV, known fibrotic markers (Qian et al., 2016). Consistent with this, it was demonstrated *in vivo* that proteinase-activated receptor-2 (PAR2) activation in kidney tubular epithelial cells transactivates the EGF and TGFβ receptors. This in turn resulted in Smad2 activation and production of the pro-fibrotic factors and CTGF, all of which contribute to renal fibrosis (Chung et al., 2013).

Supported by murine model evidence, it was concluded that tissue fibrosis resulting from aldosterone depends on ROS-induced EGFR/ERK activation, thus making EGFR a possible point for controlling renal fibrosis (Sheng et al., 2016). Likewise, continuous activation of EGFR by expressing the EGFR ligand human heparin-binding EGF-like growth factor (hHB-EGF) in the proximal tubule epithelium of a mouse model kidney gave rise to spontaneous, progressive renal tubulointerstitial fibrosis (Overstreet et al., 2017). To study the methods of albumin-induced renal fibrosis, it was observed that EGFR has a significant role in albumin-induced fibrotic events, as inhibition of γ-secretase activity played the major role, depending on ERK-MAPK (Slattery et al., 2013). The ligands responsible for this sustained EGFR activation were suggested to be Src mediated, which is needed in the stimulation of renal interstitial fibroblasts and gene expression of numerous profibrogenic cytokines, such as TNF-b1 (TGF-b1) (Tang et al., 2013b). To evaluate the role of Src, PP1 (a selective inhibitor of Src) was introduced into cultured renal interstitial fibroblasts (NRK-49F). PPI treatment inhibited stimulation of TGF-β1 signaling, activation of EGFR and STAT3, and decreased amount of renal epithelial cells arrested at the G2/M phase of the cell cycle after ureteral obstruction (Yan et al., 2016). To support this, it was shown that renal fibrosis was decreased when Fyn (a member of the Src family of kinases) deficiency was implemented by the inhibition of phospho-STAT3 (Seo et al., 2016). Another reason for this sustained EGFR activation could be the excessive expression of connective tissue growth factor (CCN2) in fibrotic areas. It was shown that upon administration of CCN2, the fibrotic response in the kidney of a murine model was diminished by EGFR blockade, indicating that blocking EGFR could be a promising treatment option to halt the profibrotic effects of CCN2 in renal pathologies (Rayego-Mateos et al., 2018a, 2018b).

HB-EGF, another ligand for EGFR, is likely also involved in renal fibrosis. Reduced renal fibrosis was noticed when endothelial HB-EGF expression was deleted, by diminishing Ang II infusion-related renal damage (Zeng et al., 2016). It is well known that ErbB4 expression increases in CKD. A study reported that a novel long non-coding RNA (lncRNA) called Erbb4-IR is accountable for renal fibrosis that is mediated by TGF-β/Smad3, making it a selective therapeutic target for CKD (Feng et al., 2018). Supporting this, it was also found that by suppressing miR-29b, renal fibrosis was enhanced in T2DN by Erbb4-IR through a Smad3-dependent lncRNA (Sun et al., 2018).

It has been implicated that oxidative stress could lead to CKD. While studying the possible underlying mechanisms for this, it was found that CKD-bearing mice given Tempol (a Superoxide Dismutase-Mimetic) exhibited reduced renal fibrosis along with decreased signaling via TGF-ß/Smad3, EGFR and MAPK pathways (Ding et al., 2015). Furthermore, exploring other possible mechanisms, it was found that EGFR activation, p53, and ROS are all needed in the initiation of renal fibrotic genes by TGF-β1, confirming the role of EGFR in renal fibrosis (Samarakoon et al., 2013).

Studying the precise EGFR signaling cascades, it was proven that mice lacking ADAM17 in smooth muscle cells had transient protective effects from renal fibrosis (Shen et al., 2017). These results come in line with those that indicated that the hepatic stellate cell (HSC) transactivation of Ang II-induced EGFR is mediated through ADAM17, which could lead to the progression of liver fibrosis (Oikawa et al., 2014). Histone deacetylases classes (HDAC) 1 and 2 were comprehensively investigated for their part in the stimulation of fibroblasts and the pathogenesis of numerous chronic diseases, including renal fibrosis (Marchion and Münster, 2007; Liu et al., 2013; Wang et al., 2022). Furthermore, it was found that renal fibrosis improved along with attenuation of renal fibroblast activation by manipulating TGB-beta and EGFR signaling through blocking class I histone deacetylase (Liu et al., 2013). Blocking Sirtuin-1 and 2 (Class III HDAC) prevented renal interstitial fibroblast activation and halted renal interstitial fibrosis in obstructive nephropathy through inhibition of EGFR and PDGFRβ signaling (Ponnusamy et al., 2014). A recent study of a unilateral ureteral obstruction (UUO) mouse, the expression of an E3 ubiquitin ligase called HUWE1 and EGFR was examined. Importantly, the overexpression of HUWE1 decreased the expression of EGFR, by physically interacting with the receptor and promoting its ubiquitination and degradation, hence regulating the expression of EGFR, and protecting against kidney injury (Zhu et al., 2020).

Inhibiting AREG, a ligand of EGFR, by AREG-targeting Self-Assembled-Micelle inhibitory RNA (SAMiRNA-AREG) in animal models of renal fibrosis resulted in decreased expression of fibrotic markers, including α-smooth muscle actin, fibronectin, α1(I) collagen, and α1(III) collagen. Remarkably, reduced renal fibrosis was accompanied by decreased EGFR phosphorylation (Son et al., 2021). The aforementioned studies are therefore suggestive of an important role of EGFR in mediating renal fibrosis and its selective inhibiton might represent a viable therapeutic strategy (for a summary of selected studies see also Table 7).

**TABLE 7:** Summary of selected studies highlighting the role of EGFR signalling in renal fibrosis.

Number	Model	Methodology	Findings/Results/Conclusion relevant to EGFR	Study Reference
1	Mouse models and Human CKD patients	Unilateral ureteral obstruction induced renal fibrosis in T1D and T2D nephropathies	Significant increase in HER2	Li, H et al. (2019)
2	Mice model	Erlotinib action on CKD rats with remnant kidney	Inhibiting Akt and ERK 1/2 signaling resulted in reduced levels of cortical profibrogenic genes along with glomerulosclerosis and tubulointerstitial damage	Yamamoto et al. (2018)
3	Murine model	EGFR mimotope in unilateral ureteral obstruction	Improved renal fibrosis, decreased expression of collagen I, fibronectin and α-SMA	Yang et al. (2019)
4	Mice model	Ang II-stimulated mice treated with EGFR inhibitor 453	Inhibition of AKT and ERK pathways 453 also inhibited the activation of fibrotic pathways such as collagen, CFGF, TGF-β	Skibba et al. (2016)
5	Mice model	Usage of short hairpin RNA knockdown to inhibit the activation of EGFR in Angiotensin II-treated SV40 mesangial cells	Abolished buildup of fibrotic markers including CTGF and collagen IV	Qian et al. (2016)
6	Mice model	PAR2 activation in kidney tubular epithelial cells	Enhancement of Smad2 activation and release of pro-fibrotic factors via transactivation of EGF and TGFβ	Chung et al. (2013)
7	Murine model	Erlotinib treated aldosterone-mediated tissue fibrosis mice	TGF-β, α-SMA, and mesangial matrix proteins including collagen Ⅳ and fibronectin had inhibited expression	Sheng et al. (2016)
8	Mouse model	EGFR activation by expressing EGFR ligand hHB-EGF	Evolvement of progressive renal tubulointerstitial fibrosis	Overstreet et al. (2017)
9	Human HK-2 cell line	Incrementing concentrations of albumin on fibrosis indicators and mediators in models with and without γ-secretase inhibitor	EGFR has a major role in albumin-induced fibrotic events via halting γ-secretase activity	Slattery et al. (2013)
10	Murine model	PP1 treating cultured renal interstitial fibroblasts	Src inhibition prevented activation of signaling pathways including TGF- β1, EGFR and STAT3, ameliorating renal fibrosis	Yan et al. (2016)
11	Mice model	Unilateral ureteral obstruction resulting in renal fibrosis	Decreased renal fibrosis was found in Fyn-deficient mice, by inhibition of phosphor-STAT3	Seo et al. (2016)
12	Murine model	Administration of CCN2 with EGFR blockade	Decreased fibrotic response in kidney	Rayego-Mateos et al. (2018a)
13	Mice model	Ang II with endothelial HB-EGF deletion	Compared with controls, decreased EGFR activation and diminished renal fibrosis	Zeng et al. (2016)
14	Mouse model	Using a Smad3-dependent mechanism, Erbb4-IR induced by TGF-β1 was silenced	Reduced renal fibrosis along with blockage of collagen I and α-SMA expressions that are TGF-β1-induced *in vitro*	Feng et al. (2018)
15	Mouse model	Silencing Erbb4-IR induced by T2DN	Protecting the kidney in db/db mice models from progressive renal injury due to amplified miR-29b	Sun et al. (2018)
16	Mice model	Tempol-treated CKD mice	TGF-ß/Smad3-induced renal fibrosis along with EGFR/MAPK pathways activation was inhibited	Ding et al. (2015)
17	Mice model	ADAM17 lacking Ang II-infused mice	*In vivo* transient protective effects from renal fibrosis	Shen et al. (2017)
18	Murine model	HDAC 1 blockage in unilateral ureteral obstruction mice	Modulation of TGF-beta and EGFR signaling resulted in amelioration of renal fibrosis and inhibition of the activation of renal fibroblasts	Liu et al. (2013)
19	Mouse model	HDAC 3 blockage in renal interstitial fibrosis model of obstructive nephropathy	Attenuation of renal interstitial fibrosis	Ponnusamy et al. (2014)
20	C57BL/6J mice	Expressing of EGFR along with HUWE1 in a UUO mice model	HUWE1 regulated EGFR expression and resulted in a protective effect against kidney injury	Zhu, Q et al. (2020)
21	C57BL/6 mice	Renal fibrosis generated by a UUO and adenine diet (AD) investigating SAMiRNA-AREG effect on the disease	SAMiRNA-AREG suppressed EGFR signals and is considered as a new siRNA treatment for renal fibrosis	Son et al. (2021)

## 9 Potential cross talk between EGFR/ErbB family of receptors and Ang-(1–7) in renal pathologies

The renin-angiotensin-aldosterone system (RAAS) plays a key role in regulating cardiovascular homeostasis. For many years Ang II which is a pro-hypertensive peptide that is known to have inflammatory and fibrotic effects was regarded as the only active member of the RAS. However recent studies have shown that Angiotensin-(1–7) [Ang-(1–7)] is also an active part of RAAS and produces effects that are opposite to those of Ang II (Benter et al., 1993; Benter et al., 1995a; Benter et al., 1995b). Today, it is well accepted that RAAS has two opposing arms with their relative balance determining the precise physiological and pathological consequences of RAAS (Benter et al., 2006; Benter et al., 2007; Ferrario et al., 2010; Ferrario et al., 2005; Li et al., 2017; Bader et al., 2024; Ye et al., 2004).

Activation of the ACE-Ang II-AT1 receptor arm produces pro-inflammatory, pro-oxidative stress, and pro-fibrotic signals leading to cardiovascular disorders. These pathophysiological effects are known, in part, to occur via Ang II mediated transactivation of EGFR and/or other ErbBs (Lautrette et al., 2005; Akhtar et al., 2012; Akhtar et al., 2015; Forrester et al., 2018; Akhtar et al., 2020; Shraim et al., 2021). Overactivity of this arm has been implicated in various pathologies, including hypertension, cardiovascular diseases, diabetes-induced complications, respiratory and renal diseases (Ferrario et al., 2010; Ferrario and Varagic, 2010; Re RN, 2018; Paz Ocaranza et al., 2020). This over activity can be mitigated by RAAS inhibitors such as ACE inhibitors (ACEIs), AT1R blockers (ARBs), and mineralocortocoid receptor inhibitors (MCRIs), commonly used in managing hypertension, heart failure, cardiac myopathies, and delaying the progression of cardiac/renal pathologies (Arendse et al., 2019) as well as by EGFR inhibitors (Qian et al., 2016; Rintala et al., 2016).

In the counter-regulating arm, ACE2 converts Ang II to Ang-(1–7), which acts via its G-protein coupled Mas receptor (MasR) (Kostenis et al., 2005). Ang-(1–7) inhibits ACE activity and decreases Ang II production whereas MasR is known to heterodimerize with AT1 receptor and inhibit actions of Ang II (Kostenis et al., 2005; Galandrin et al., 2016; Teixeira et al., 2017). Activation of the ACE2-Ang-(1–7)-MasR arm leads to vasodilation, anti-proliferative, anti-inflammatory, anti-oxidative stress, and anti-fibrotic effects (Benter et al., 1993; Benter et al., 1995a; Benter et al., 1995b; Dhaunsi et al., 2010; Ferrario et al., 2010). Indeed, Benter and colleagues were the first to report that Ang-(1–7) behaves differently from Ang II and produces vasodilatory effects by activating its own receptor in a pithed rat model in 1993 that was later confirmed in animal models of hypertension (Benter et al., 1993; Benter et al., 1995a; Benter et al., 1995b). In human subjects, Ang-(1–7) produces vasodilation in the coronary, splanchic, and renal circulations (Schindler et al., 2007; Medina and Arnold, 2019). Numerous studies have shown that ACE2-Ang-(1–7)-MasR activation is protective in various disease states that include renal, cardiovascular, respiratory, and diabetes-induced complications (Ferrario et al., 2005; Benter et al., 2006; Benter et al., 2007; Benter et al., 2008; Ferrario et al., 2010; Ferrario and Varagic, 2010; El-Hashim et al., 2012; Benter et al., 2015; Cao et al., 2019; Santos et al., 2019; Magalhães et al., 2020).

The kidney is a key site for Ang-(1–7) production and ACE2 protein, which is expressed in glomerular endothelial cells, podocytes and proximal tubular endothelial cells, and has been reported to be decreased in kidneys from diabetic and hypertensive mice (Crackower et al., 2002; Tikellis et al., 2003; Mizuiri and Ohashi, 2015). Furthermore, deletion or suppression of the ACE2 gene is associated with renal injury, dysfunction, and fibrosis (Ortiz-Melo and Gurley, 2016) and more severe nephropathy in response to high blood pressure (Liu et al., 2017). Conversely, treatment with exogenous recombinant ACE2 or transgenic overexpression of ACE2 has been demonstrated to limit kidney injury (Ortiz-Melo and Gurley, 2016). Overexpression of human ACE2 in a model of kidney injury showed a protective phenotype (Wang et al., 2015). Furthermore, administration of Ang-(1–7) has been shown to reduce renal resistance and improve renal vascular function in rats (Benter et al., 2008). In rats with diabetic nephropathy, Ang-(1–7) has been shown to reduce proteinuria and improve kidney function (Benter et al., 2007; Kim et al., 2015; Shi et al., 2015). In hypertensive kidney disease, Ang-(1–7) plays a protective role independent of its effects on blood pressure (Benter et al., 2006; Chen et al, 2019). Ang-(1–7) also protects against drug (Adriamycin)-induced nephropathy and renal ischemia-reperfusion injury in animal models (Silveira et al., 2013; Zaman and Banday, 2022). In humans, levels of Ang-(1–7) are reduced in patients with chronic kidney disease, and supplementation with Ang-(1–7) may improve kidney function in these patients (Chen and Harris, 2016).

Ang II activation of the AT1R contributes to proximal tubule brush border injury and remodeling, in part, due to enhanced mTOR signaling which promotes tubulointerstitial fibrosis (Whaley-Connell et al., 2011). Studies in models of diabetic and polycystic kidney disease indicate that targeting reductions in mTOR activity attenuates the progression of tubulointerstitial fibrosis (Whaley-Connell et al., 2011). AT1R antagonist telmisartan was shown to reduce the phosphorylation of EGFR and ERBB2 in human esophageal adenocarcinoma cells and inhibit cell proliferation and tumor growth, inducing cell cycle arrest by regulating cell cycle-related molecules via the AMPK/mTOR pathway (Fujihara et al., 2017). It has been shown that ACE inhibitors and ARBs increase Ang-(1–7) formation and Ang-(1–7) receptor antagonists attenuate their cardiovascular benefits indicating that at least part of the beneficial effects of ACEIs and ARBs are due to Ang-(1–7) activation (Ishiyama et al., 2004; Benter et al., 2011; Santos et al., 2019). Recently it was shown that Ang-(1–7) suppresses mTOR signaling, mitigates oxidative stress and protects human proximal tubular cells from endoplasmic reticulum stress, mitochondrial dysfunction and apoptosis (Farooqui and Banday, 2024). Although some opposing views exist (Zimmerman and Burns, 2012), the majority of studies provide increasing evidence for the renoprotective effects of ACE2/Ang-(1–7)/Mas receptor pathway that most likely occurs, at least partly, through counteracting the actions of ACE/Ang II/AT1 arm of RAAS and EGFR signalling.

So how might Ang-(1–7) precisely function in ameliorating renal pathologies? Oxidative stress and inflammation are well-known drivers of kidney injury, and reducing these factors is a key strategy in the prevention and treatment of kidney disease. The potential benefits of Ang-(1–7) in the regulation of kidney function are believed to be due to its ability to reduce oxidative stress, inflammation and the ensuing fibrosis in the kidney, as well as to its direct effects on renal blood flow and function (Benter et al., 2008; Lu et al., 2017; Yousif et al., 2012; Simões e Silva et al., 2013). Ang-(1–7) has been shown to have direct anti-inflammatory and antioxidant effects in the kidney (Benter et al., 2008; Shi et al., 2015). In a study by Lu et al. (2017), Ang-(1–7) was shown to reduce oxidative stress and inflammation in an experimental model of renal injury. Additionally, administration of Ang-(1–7) has been shown to improve renal vascular function and to improve kidney function in rats (Benter et al., 2007; Van Twist et al., 2014; Kim et al., 2015; Shi et al., 2015). These effects are believed to be due to the ability of Ang-(1–7) to reduce renal resistance, promote vasodilation, and increase blood flow to the kidney-characteristics that are actually exacerbated by EGFR signaling. Therefore, could the beneficial effects of ACE2/Ang-(1–7)/Mas receptor pathway in renal pathologies be somehow mediated by inhibition of EGFR? Indeed, there now appears to be significant evidence for cross-talk between Ang-(1–7) and EGFR. We were the first to show that Ang-(1–7) inhibits EGFR transactivation *in vitro* and *in vivo* (Akhtar et al., 2012). More recently, it was shown that Ang-(1–7) also inhibits transactivation to ErbB2, ErbB3 and ErbB4 receptors from the EGFR family of receptor tyrosine kinases and might act as a novel “pan-ErbB inhibitor (Akhtar et al., 2015). Furthermore, it was shown in the diabetic vasculature that Ang-(1–7)-mediated inhibition of EGFR receptor family occurred via a src-dependent pathway (Akhtar et al., 2012; Akhtar et al., 2015). As to whether this occurs in the diabetic kidney pathologies remains unknown. However, in a study by Qin et al. (2015), administration of Ang-(1–7) reduced EGFR expression and activity in the kidney in rats with experimental glomerulonephritis supporting the notion that Ang-(1–7) is an inhibitor of EGFR and that this might represent a potential mechanism by which it exerts its beneficial renal effects.

Overall, the potential interplay between EGFR and Ang-(1–7) in the kidney is likely to be complex and multifaceted relationship depending on the site and duration of EGFR actions. Thus, further research is needed to fully understand the mechanisms involved. However, the current evidence suggests that both EGFR and Ang-(1–7) are important effectors in the regulation of kidney function, and that the interplay between these two systems may play a key role in the prevention and treatment of kidney disease.

## 10 Concluding remarks

Our review paper highlights the role of epidermal growth factor receptor (EGFR) and its downstream signaling in seven different kidney pathologies: glomerulonephritis, diabetic nephropathy, hypertensive nephropathy, acute kidney injury, Chronic kidney disease, real transplant, and renal fibrosis. EGFR signaling appears to have a dual role in kidney physiology, repair and pathology. Although EGFR activation is crucial for kidney physiology and repair such as following acute kidney injury through mechanisms involving dedifferentiation, migration and proliferation of tubular cells, its sustained activation has been linked to renal fibrosis via activation of myofibroblasts. The overall impact of EGFR activation on kidney health depends on the duration and site of activation. Where overactivity of EGFR family members has been demonstrated, pre-clinical studies with inhibitors of EGFR family of RTKs as potential therapeutic agents have shown promising results in some kidney pathologies. Indeed, small molecule EGFR inhibitors such as Gefitinib and Erlinotib have been clinically used in cancer chemotherapy since their FDA approval in the early 2000s (Karachaliou et al., 2019; Abourehab et al., 2021; Singh et al., 2023; Zubair and Bandyopadhyay, 2023). They are deemed to have an acceptable safety profile with diarrhea and skin rashes as the most common side effects but these can be readily managed with conventional approaches (Hirsh, 2011; Aw et al., 2018). Relatively more recently, large molecule, monoclonal antibody-based inhibitors of EGFR family of receptors, such Cetuximab and Panitumumab, have also been introduced clinically for the treatment of several cancers (Yamaoka et al., 2017). The clinical effectiveness and seemingly acceptable safety and toxicological profile of both large and small molecule inhibitors suggests that the repurposing of these FDA-approved anticancer EGFR inhibitors for renal pathologies might be a clinically feasible therapeutic approach-though this requires further clinical evaluation including monitoring the possible development of drug resistance (Cooper et al., 2022). Thus, it is clear that further research is necessary to fully understand the role of these RTKs, including their interplay with members of the RAAS such as Ang-(1–7) as well as the potential use of specific EGFR inhibitors, in renal pathologies in the clinical setting.
